# External femoral rotation of 2 degrees is associated with the lowest stuffing rates around the femur in functionally aligned TKA

**DOI:** 10.1002/ksa.12662

**Published:** 2025-03-28

**Authors:** Alexander Maslaris, Eustathios Kenanidis, Nikolaos Mylonakis, Zakareya Gamie, Abtin Alvand, William F. Jackson, Andrew J. Price, Eleftherios Tsiridis

**Affiliations:** ^1^ Academic Orthopaedic Department Aristotle University Medical School, General Hospital Papageorgiou Thessaloniki Greece; ^2^ Nuffield Orthopaedic Centre, Oxford University Hospitals NHS Trust Oxford UK

**Keywords:** femoral rotation, overstuffing, patellofemoral joint, personalized total knee arthroplasty, trochlea anatomy, understuffing

## Abstract

**Purpose:**

Modifying femoral morphology after total knee arthroplasty (TKA) poses a potential risk for ligament‐imbalances and patella mal‐tracking. The purpose of this study was primarily to quantify TKA‐induced stuffing around the femur and secondarily assess the effect of femoral rotation (FR) on trochlear and condylar anatomy‐changes.

**Methods:**

Knee anatomy‐modification was quantified in 69 robotic‐assisted TKAs utilising tibia‐based functional alignment (FA). Caliper‐measurements were performed on the medial (Med), central (Ctr), and lateral (Lat) sides of the following resection planes: anterior trochlea (AT), oblique trochlea (OT), distal condyles (DC), posterior condyles (PC) and tibia (TIB). The same caliper‐measurements were performed on the femoral components used to calculate bone‐implant differences and analyse possible patterns of postoperative trochlear anatomy‐modifications (TAM) and condylar anatomy‐modifications (CAM). Over‐ or understuffing analysis for different FRs and regression analysis were conducted to assess the effect of FR on CAM and TAM.

**Results:**

TAM results were Lat‐AT −3.2 mm ([95% confidence interval [CI]: −3.71 to −2.63], *p* < 0.001), Ctr‐AT 0.7 mm ([95%CI: 0.22–1.32], *p* = 0.02), and Ctr‐OT −1.7 mm ([95%CI: −1.85 to −0.93], *p* < 0.001) with stuffing > 2 mm in 60.9%, 39.1%, and 39.1%, respectively. CAM results were Med‐DC −3.6 mm ([95%CI: −4.14 to −3.05], *p* < 0.001) and Lat‐PC 3.0 mm ([95%CI: 2.48–3.38], *p* > 0.001) with stuffing > 2 mm in 78.3% and 63.8%. FR (3.8 ± 2.6°, range: −1.6° to 8.5°) affected mostly the anterior (*r* = −0.40, *p* < 0.001) and posterior (*r* = 0.71, *p* < 0.001) aspects of the knee but hardly the OT plane (*r* = 0.06, *p* = 0.624) and the trochlear groove to its full range of flexion (*r* = 0.21, *p* = 0.17). External FR 2° was associated with the lowest incidence of femoral stuffing > 2 mm and ≥ 4 mm.

**Conclusions:**

FA‐typical modification‐pattern was a TAM with lateral facet understuffing, and CAM with medial distal understuffing and lateral posterior overstuffing. Trochlear groove height was non‐significantly affected by FR. FA with the current off‐the‐shelf implant induces the lowest stuffing rates when set in 2° external femoral rotation.

**Level of Evidence:**

Level II.

**Abbreviations**
AT: anterior trochlea
CAM: condylar anatomy modification
cFEA: condylar flexion‐extension axis
Ctr: central
DC: distal condyle
Diff: difference (mediolateral)
DT: distal trochlea (groove)
FA: functional alignment
FR: femoral rotation
HKA: hip‐knee‐ankle angle
JLO: joint line obliquity
KA: kinematic alignment
Lat: lateral
LDFA: lateral distal femoral angle
MA: mechanical alignment
Med: medial
MPTA: medial proximal tibia angle
OA: osteoarthritis
OT: oblique (chamfer) trochlea
PC: posterior condyle
PCA: posterior condylar axis
pFEA: patellar flexion‐extension axis
PFJ: patellofemoral joint
RaTKA: robotic‐assisted TKA
SD: standard deviation
TAM: trochlea anatomy modification
TIB: proximal tibia
TKA: total knee arthroplasty

## INTRODUCTION

Personalized total knee arthroplasty (TKA) is an ongoing evolving process [10, 19, 20, 56]. Rapid development of technology in TKA and the introduction of robotic‐assisted surgery has enhanced orthopaedic procedures by improving precision and accuracy in component positioning [17, 26, 31, 38].

**Figure 1:**
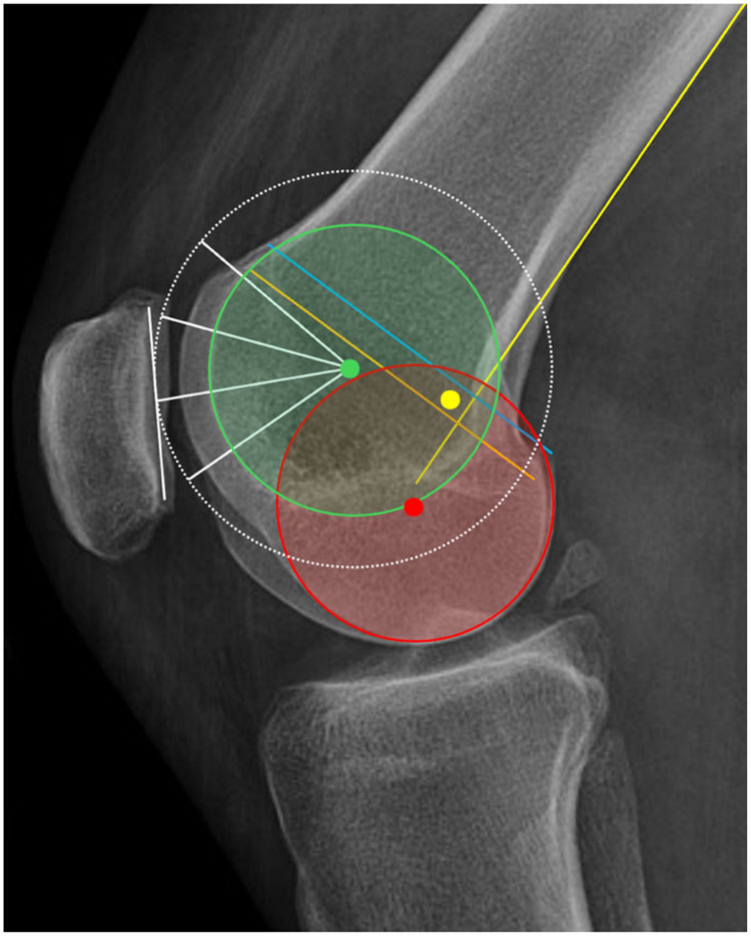
Flexion‐extension axis of the femorotibial joint (*tibial articular surfaces* of the femoral condyles) in red colour. Trochlea groove radius and axis (*patellar articular surfaces* of the femoral trochlea) in green colour. The flexion‐extension radii of the patella in white colour with its centre fitting very closely the trochlea groove axis (in this case they are equal but may vary between different trochlea forms). The *Schoettle point* in yellow colour is located more posterior and proximal to the groove centre: Posterior femoral cortical line (yellow), posterior femoral condylar line (blue), posterior point of Blumensaat line (orange).

Any compromise of the normal native femoral anatomy can cause dysfunction of both the tibiofemoral (TFJ) and patellofemoral joints (PFJ) [1, 13]. Changes of the femoral form alters the orientation of the condylar flexion‐extension axis (cFEA) and the patellar flexion‐extension axis (pFEA) resulting in imbalances of the TFJ and PFJ [30, 32] (Figure 1). Stuffing of the anterior or posterior femoral surfaces > 2 mm affects the PFJ biomechanics by changing the lever arm of the extensor mechanism, and the patella‐engaging articulation of the trochlea facets [13, 41, 68]. Stuffing the distal surface may affect the PFJ in flexion which may cause anterior knee pain. Trying to balance the flexion gap by changing femoral rotation (FR) will alter the anterior space affecting the PFJ in extension.

There is limited evidence about the magnitude of changes that occur around the femur after personalised TKA. Clinical studies focus on the reconstruction of the femoral condyles and not on the trochlea [6]. Analyses on trochlea changes after TKA rest solely upon image‐based reconstructions [36, 52, 54] or data retrieved from robotic software [28, 62, 63] and X‐ray measurements using various techniques [29, 48].

The aim of this study was primarily to assess different pattern of trochlear and condylar anatomy modifications using real‐time caliper measurements and to examine the incidence of stuffing > 2 mm and ≥4 mm on 11 different areas around the femur utilising functional alignment. Particular focus was given on the trochlear flanges and groove to its full range of flexion. Our secondary aim was to examine the effect of FR on the caliper measurements and the incidence of stuffing around the knee.

We hypothesised that first, there would be a knee anatomy modification with stuffing > 2 mm in more than 40% of the trochlear articular surface [62] and the condylar articular surface. Second, FR will affect stuffing mostly on the anterior and posterior aspects of the knee but not on the trochlear groove, and third, 0° FR to the posterior condylar line will induce the lowest incidence of femoral stuffing > 2 mm or ≥4 mm.

## MATERIALS

### Patient recruitment

This is a single‐centre prospective cohort study. Consecutive patients that were treated with TKA from July 2023 to April 2024 for end‐stage osteoarthritis (OA) of the knee were recruited. All patients received an imageless robotic‐assisted total knee arthroplasty (ROSA® Knee System, Zimmer Biomet) using the same compatible posterior‐stabilised implant in all times (﻿LPS Flex precoat, NexGen Legacy, Zimmer Biomet, Warsaw, IN).

Our study had the following exclusion criteria: Revision TKA, TKA performed for indications other than OA, TKA not performed using functional alignment. Further data exclusions were made from caliper measurements due to poor bone quality or undefined recuts and cases with missing caliper measurements. Cases with inadequate pre‐operative X‐rays causing projection‐related measurement errors and measurements from patients that refused participating in the study were not included.

A functional alignment protocol [61] was used in all cases to adjust 3D implant position aiming for balanced gaps, individualised bony cuts close to the constitutional joint line obliquity, restoring the pre‐arthritic limb axis and limiting soft tissue releases. The surgical technique is described in previous studies [12]. Utilising MA‐landmarks as a starting reference point, coronal boundaries of ±5° for restoration of the joint line obliquity and HKA were used. The definitive implant position was adjusted according to surgeon's preferences to achieve proper gap balance even if individual cases exceeded these predefined zones. After primarily setting the desired tibia component orientation (tibia‐based technique), the femur could be then reconstructed accordingly.

### Data analysis

All bone resections were measured using a caliper to assess their thickness accounting also for cartilage loss and saw blade thickness. Recuts were added to the calculations.

Cartilage loss was simplified in three stages:

I. *Healthy cartilage* (no loss) = 2 mm healthy layer.
II. *Moderate cartilage damage* (50% loss) = 1 mm healthy layer.
III. *Severe cartilage damage* (100% loss) = 0 mm healthy layer.

**Figure 2:**
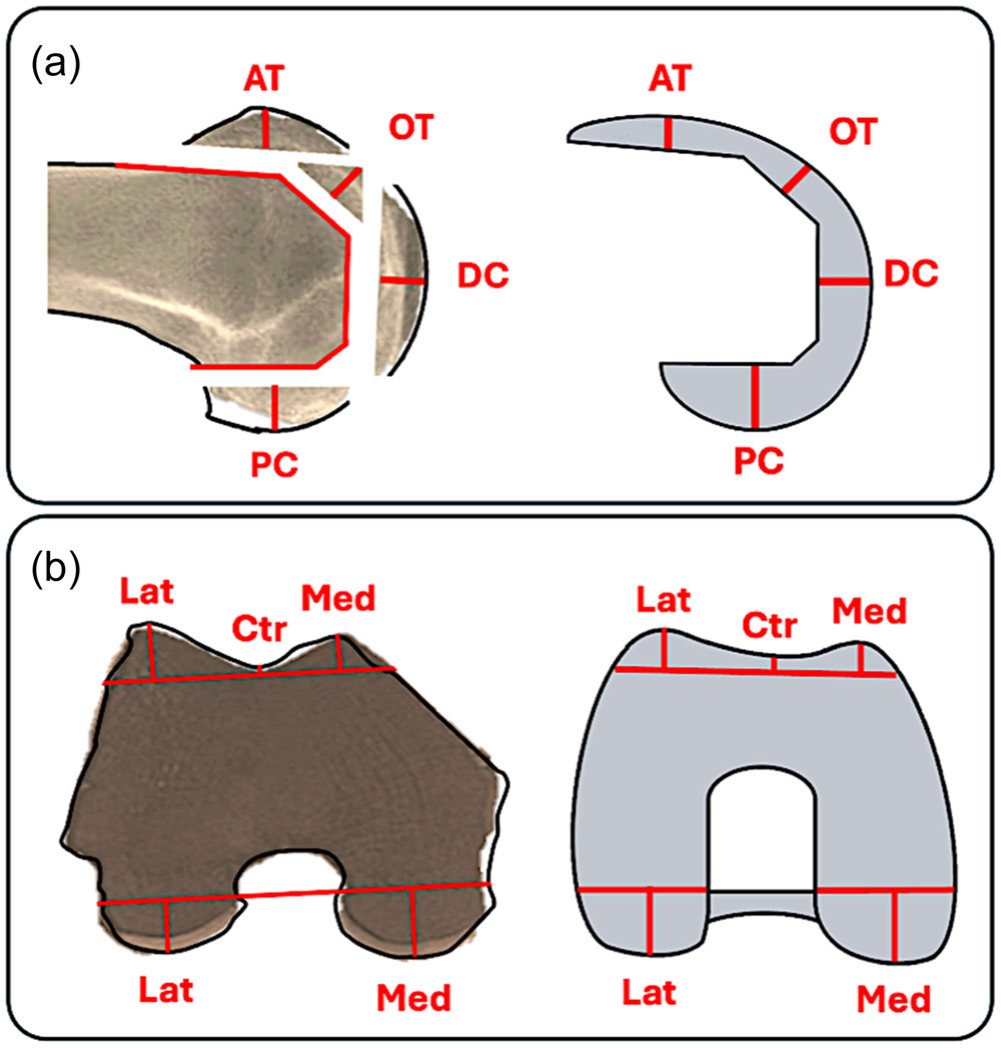
(a, b) Same areas of caliper measurements were used on both, bone resections and femoral components to assess postoperative stuffing of the articular surfaces of the knee. (a) Sagittal plane and (b) axial plane. AT, anterior trochlea; Ctr, central; DC, distal condyle; Lat, lateral; Med, medial; OT, oblique (chamfer) trochlea; PC, posterior condyle.

The following resection planes were assessed: Anterior trochlea (AT), oblique (chamfer) trochlea (OT), distal condyle (DC), posterior condyle (PC) and proximal tibia (TIB). Each of the above resection planes were measured on their medial (Med), central (Ctr) and lateral (Lat) portion when applicable. The central parts of the AT, OT and DC resection planes were the deepest points and represent the trochlear groove in full range of motion of the PFJThe same caliper measurements were than performed on the implants used to assess potential differences with the corresponding bone cut thicknesses (Figure 2a,b). Based on previous studies, changes > 2 mm were used as a threshold to define overstuffing (+) or understuffing (−) [13, 41, 53, 68]. Finally, the incidences of stuffing > 2 mm and ≥4 mm for each resection area were examined. The knee anatomy modification was than divided into (1) condylar anatomy modification (CAM) and (2) trochlea anatomy modification (TAM) as each area represents a different articular space and a different flexion‐extension axis (Figure 1).

Assessing the overall incidence of femoral stuffing involved calculation of absolute differences between the components and bony measurements. The overall femoral stuffing analysis was conducted separately for (a) the femur (all measurements of AT, OT, DC and PC), (b) the trochlear medial margin (medial measurements of AT, OT and DC), (c) the trochlear groove (central measurements of AT, OT and DC), and (d) the trochlear lateral margin (lateral measurements of AT, OT and DC). Each analysis‐group was then divided in following subgroups of FR to assess degree‐wise differences in the incidences of stuffing: <1°, 1°, 2°, 3°, and ≥4°.

Calliper measurements of the femoral resected specimens were made twice from two different observers independent from one another to assess the interobserver agreement. Interclass correlation coefficient (ICC) was conducted to report the kappa values according to the criteria by Landis and Kock [35].

Following alignment values were retrieved intraoperatively from the Rosa database: (1) limb alignment initial and after TKA, (2) tibia cut obliquity (in relation to mechanical axis), (3) tibial slope, (4) femoral rotation (in relation to posterior condylar axis) and (5) femoral flexion.

X‐rays were used for the measurement of the lateral distal femoral angle (LDFA) and the medial proximal tibia angle (MPTA) to assess their preoperative‐to‐postoperative difference and therefore the degree of surgery‐related coronal alignment changes of the knee. Coronal plane alignment of the knee (CPAK) [37] and the functional knee phenotypes (FKP) [21] were both calculated before and after surgery to assess alignment type distribution changes in order to account for variation in knee phenotypes between individuals [7, 11, 20].

Positive values (+) represented the following: Valgus limb alignment (>180°), valgus MPTA (>90°), varus LDFA (>90°), externa FR and femoral extension. Negative values (−) represented: Varus limb alignment (<180°), varus MPTA (<90°), valgus LDFA (<90°), internal FR and femoral flexion.

Measurements were expressed in mean ± standard deviation (SD) and range (Min to Max).

To observe the relation between FR of the FA technique and the resection thicknesses (around the knee and therefore the stuffing, scatter plots with liner regression analysis were used. Using the corresponding implant thickness as a reference line and a ±2 mm “balanced zone” above and below it, the overstuffing or understuffing tendances could be illustrated.

IRB approval was obtained from the faculty of Health Sciences, Medical Department, Bioethics and Ethics Committee, Aristotle University Thessaloniki, Greece ﻿(IRB No. 4/13.02.2024).

### Statistical analysis

To determine sample sizes and statistical power for each hypothesis of the current study a power analysis (G*Power 3.1) was conducted: For the simple linear regression analysis using one predictor (femoral rotation), a power of 0.8, a‐error of 0.05 and a medium effect size *f* ^2^ of 0.15 recommended a sample size of *n* = 55. For the comparison of the caliper measurements between resections and implant thickness the effect size was calculated based on previous literature: [63] Anticipating a mean trochlea groove stuffing of 0.8 mm ± 1.2 mm after RaTKA in FA technique when compared with 1.36 mm ± 1.2 mm (control) a medium effect size *f* ^2^ of 0.5 could be determined indicating a sample size of *n* = 64 for each group, and when aiming to detect stuffing 2 mm ± 1 mm a minimum size of *n* = 15 was recommended (actual power 0.82).

Statistical analyses were performed using IBM SPSS Statistics™ (Version 29) software. Linear regression analysis was used to assess the relationship between FR and bony resections. To compare the calliper measurements before and after surgery we used the paired *t*‐test or Wilcoxon signed‐rank test. Correlation analysis was conducted using the Pearson's correlation coefficient in normally distributed variables, otherwise the Spearman's test was used. Correlation strength was labelled according to Quilford's classification [15]. Data were tested for normality using the Shapiro‐Wilk method. *p*‐Values of <0.05 were considered statistically significant and were accompanied by an 95% confidence interval (95% CI).

## RESULTS

From the 85 consecutive cases that were recruited, 16 were excluded due to missing calliper measurements (14) and cases done with manual instrumentation (2). Therefore, 69 robotic‐assisted total knee arthroplasties utilising a FA protocol met inclusion criteria for the study, resulting in a total of 759 bone resections around the femur available for stuffing analysis.

**Figure 3:**
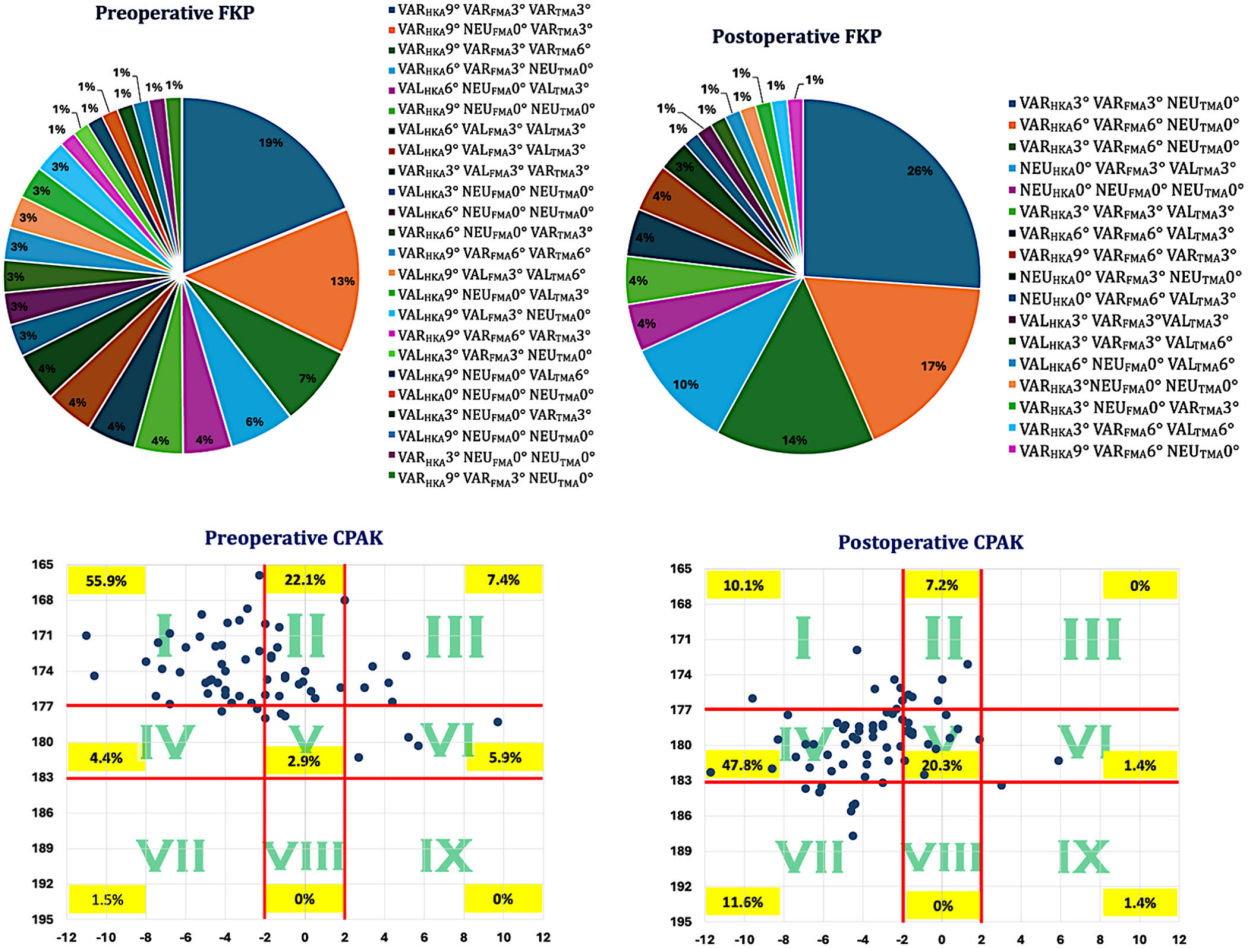
Distribution of the FKP and CPAK phenotypes before and after surgery. CPAK, coronal plane alignment of the knee; FKP, functional knee phenotypes.

The mean preoperative limb alignment was −7° ± 4.7° varus (range: −17.9° varus to 6.7° valgus) and the postoperative −4° ± 3.3° varus (range: −8° varus to 2.8° valgus). The preoperative and postoperative distribution of the FKP and CPAK phenotypes is depicted in Figure 3. While limb alignment changes remained within the same CPAK phenotypes, there was a trend in JLO change from proximal apexed to more neutral apexed phenotypes. The postoperative LDFA changed by +2.5° ± 3.1° into more varus and the MPTA by +2.4° ± 2.7° into more valgus. Mean FR was 3.8° ± 2.6° (range: −1.6° internal FR to 8.5° external FR) with 23.2% having FR < 2° and 76.8% FR ≥ 2°. The femoral coronal cut, and femoral flexion were 2.3° ± 1.6° varus (range: −2° valgus to 7.1° varus) and −2° ± 0.9° flexion (range: −3.6° flexion to 0.2° extension) respectively. The tibial coronal cut, and slope were −2.7° ± 1.7° varus (range: −6.7° varus to 1.3° valgus) and −6.1° ± 2.2° (range: −0.4° to −8.7° posterior slope).

### Main TAM and CAM outcomes

The interclass correlation coefficient (ICC) showed a good to excellent interobserver reliability (ICC 0.86–0.98) between the two observers. The implant thicknesses of the femoral component sizes used are shown in Table 1. The trochlear measurements, condylar measurements and the postoperative anatomy modifications are summarised in Table 2.

**Table 1:** Caliper measurements of the femoral component thicknesses for each implant size used.

Implant sizes	Implant thicknesses measured via caliper (mm)									
	Anterior trochlea			Oblique trochlea			Distal condyle		Posterior condyle	
	*Med*	*Ctr*	*Lat*	*Med*	*Ctr*	*Lat*	*Med*	*Lat*	*Med*	*Lat*
C	6.0	4.5	6.5	7.5	4.0	7.5	9.0	9.0	13	13
D	6.0	4.5	6.5	7.5	4.0	7.5	9.0	9.0	13	13
E	6.0	4.5	6.5	7.5	4.0	7.5	9.0	9.0	13	13
F	6.5	5.0	7.0	8.0	4.5	8.0	9.0	9.0	13	13

Abbreviations: Ctr, central; Lat, lateral; Med, medial.

**Table 2:** Bone‐implant thickness differences in millimetre (mm) based on caliper measurements.

			Bone resection thickness		Implant thickness		BID			
Variables			Mean ± SD	Range (min to max)	Mean ± SD	Range (min to max)	Mean ± SD	Range (min to max)	*p*‐value	[95% CI]
TAM	*AT*	*Med*	6.1 ± 2.5	11 (0 to 11)	6.2 ± 0.3	0.5 (6 to 6.5)	−0.2 ± 3.4	15 (−8 to 7)	*n.s*.	[−0.58, 0.79]
		*Ctr* *	4.1 ± 2.1	9 (0 to 9)	4.7 ± 0.3	0.5 (4.5 to 5)	0.7 ± 3.1	10.5 (−5.5 to 5)	0.02	[0.22–1.32]
		*Lat*	9.8 ± 2.2	9 (5 to 14)	6.7 ± 0.3	0.5 (6.5 to 7)	−**3.2** ± **2.4**	15 (−10 to 5)	<0.001	[−3.71, −2,63]
	*OT*	*Med*	7.6 ± 1.1	7.5 (5 to 12.5)	7.7 ± 0.3	0.5 (7.5 to 8)	0.1 ± 1.0	10 (−5 to 5)	*n.s*.	[−0.19, 0.31]
		*Ctr* *	5.9 ± 1.2	5.5 (3.5 to 9)	4.2 ± 0.3	0.5 (4 to 4.5)	−1.7 ± 2.4	10 (−5 to 5)	<0.001	[−1.85, −0.93]
		*Lat*	7.7 ± 0.9	5.5 (5 to 10.5)	7.7 ± 0.3	0.5 (7.5 to 8)	−0.04 ± 3.8	10 (−5 to 5)	*n.s*.	[−0.23, 0,21]
	*DT*	*Ctr* *	1.8 ± 2.5	7.5 (0 to 7.5)	0.0	0.0 0 (0−0)	−1.8 ± 2.5	7.5 (−7.5 to 0)	*n.s*.	[1.25–2.44]
CAM	*DC*	*Med*	12.6 ± 1.6	7.5 (7 to 14.5)	9.0	0.0 (9 to 9)	−**3.6** ± **2.3**	13 (−5.5 to 7.5)	<0.001	[−4.14, −3.05]
		*Lat*	10.0 ± 1.8	9 (6 to 15)	9.0	0.0 (9 to 9)	−1.0 ± 1.9	11 (−6 to 5)	<0.001	[−3.02, −2.07]
	*PC*	*Med*	13.2 ± 1.6	7.5 (9.5 to 17)	13.0	0.0 (13 to 13)	−0.2 ± 3.2	18 (−5 to 13)	*n.s*.	[−0.60, 0.25]
		*Lat*	10.1 ± 1.6	7.5 (6 to 13.5)	13.0	0.0 (13 to 13)	**3.0** ± **2.3**	18 (−5 to 13)	<0.001	[2.48–3.38]

*Note*: Bold values indicate differences >2 mm.

Abbreviations: AT, anterior trochlea; *BID*, bone‐implant thickness difference; *CAM*, condylar anatomy modification; *CI*, confidence interval; *Ctr*, central; DC, distal condyle; DT, distal trochlea (groove); *Lat*, lateral; *Med*, medial; *n.s*, nonsignificant; OT, oblique trochlea; PC, posterior condyle; *SD*, standard deviation; *TAM*, trochlea anatomy modification.

* The trochlea groove is represented by the central parts (Ctr) of the AT, OT and DT.

The absolute values and incidences of stuffing > 2 mm and ≥4 mm for each resection area are summarised in Table 3. TAM was within the ±2 mm zone in 69.3% and within ±4 mm in 86.3% with the highest incidence of stuffing found on the lateral flange of the anterior trochlea (61%). CAM was in 46.4% and 66.7% within ±2 mm and ±4 mm, respectively, with highest incidence of stuffing > 2 mm on the medial distal condyle (78.3%).

**Table 3:** Means of absolute differences between implant thickness and resection thickness after joint replacement and the percentage (%) of the resulted stuffing >2 mm and ≥4 mm of the articular surfaces.

			BID	Incidence of surface stuffing (%)	
Variables			Mean ± SD*	>2 mm	≥4 mm
TAM	*AT*	*Med*	**2.1** ± **1.8**	37.7	15.9
		*Ctr*	1.8 ± 1.6	39.1	17.4
		*Lat*	**3.3** ± **2.1**	60.9	37.7
	*OT*	*Med*	0.7 ± 0.7	2.9	1.4
		*Ctr*	2.0 ± 1.2	39.1	10.1
		*Lat*	0.7 ± 0.6	4.3	0.0
	*DT*	*Ctr*	1.8 ± 2.5	34.8	27.5
CAM	*DC*	*Med*	**3.9** ± **1.8**	78.3	56.5
		*Lat*	1.7 ± 1.7	53.6	33.3
	*PC*	*Med*	1.4 ± 1.1	18.8	2.9
		*Lat*	**3.0** ± **1.6**	63.8	40.6

*Note*: Bold values indicate differences >2 mm.

Abbreviations: AT, anterior trochlea; *BID*, bone‐implant thickness difference; CAM, condylar anatomy modification; *Ctr*, central; *DC*, distal condyle; *DT*, distal trochlea (groove); *Lat*, lateral; *Med*, medial; *OT*, oblique trochlea; *PC*, posterior condyle; SD, standard deviation; TAM, trochlea anatomy modification.

*Mean ± standard deviation of absolute difference between implant thickness and resection thickness.

### Effect of femoral rotation

The impact of FR on the resection thicknesses around the knee is illustrated on the scatter plots in Figure 4a,d. Comparing the distributions between the different resection planes of the femur and the anterior trochlea showed the highest variation. Therefore, while FR had a strong positive correlation (*r* = 0.71, *p* < 0.001) with the posterior condylar resection plane (ML measurement difference) (Figure 4c), there was only a moderate negative correlation (*r* = −0.40, *p* < 0.001) with the anterior trochlea resection plane (ML measurement difference) (Figure 4a).

Figure 4(a–d) Relation between progressive femoral rotation and resection thicknesses around the femur: (a) anterior trochlea plane and oblique (chamfer) trochlea plane, (b) trochlear groove, (c) distal and posterior femoral condyles, and (d) proximal tibia. The green dushed line represents the implant thickness (for sizes C–E) in each plane and the light green area a ± 2 mm zone, above (understuffing) and below (overstuffing) of which changes >2 mm occurred.

**Figure:**
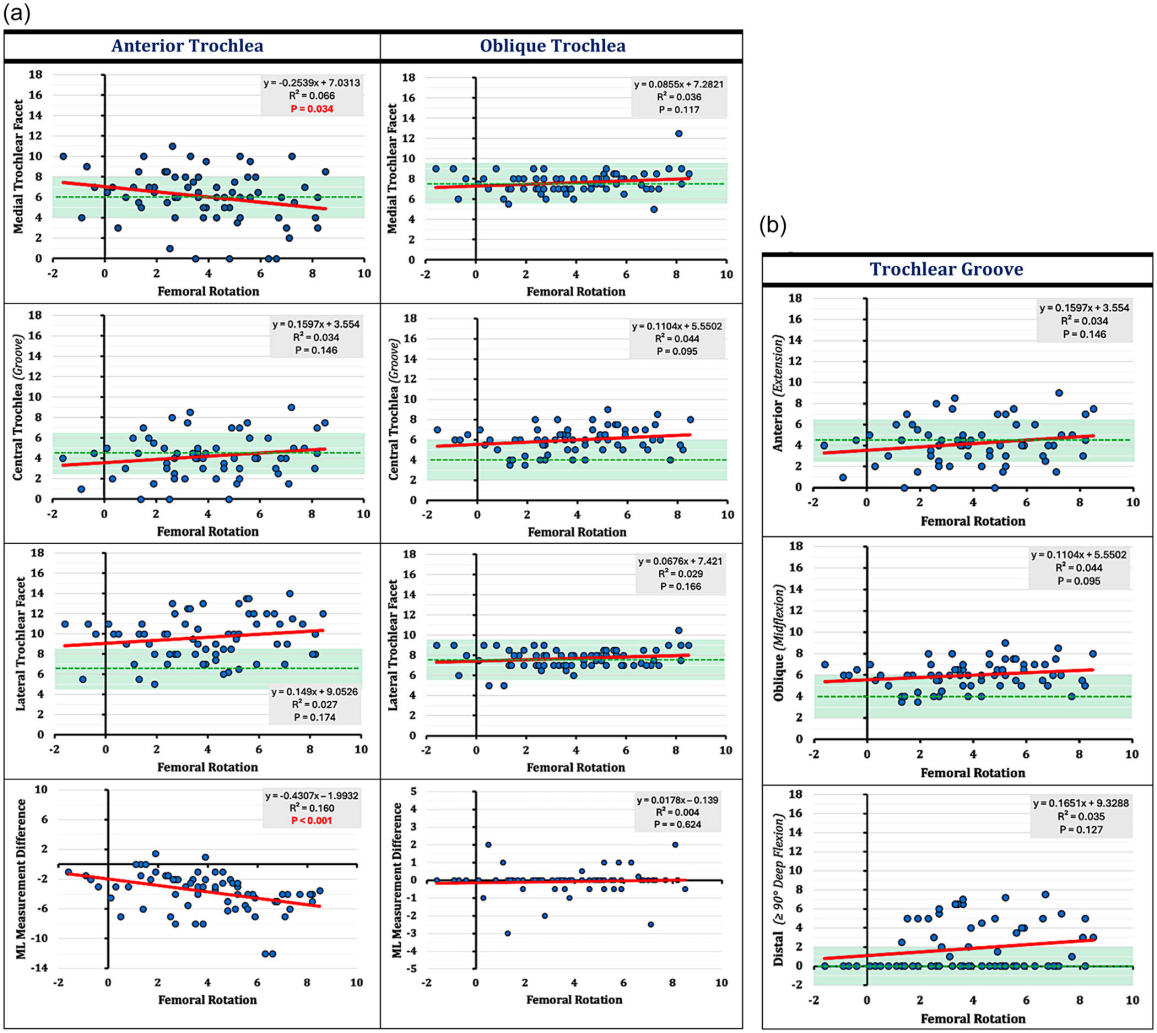

**Figure:**
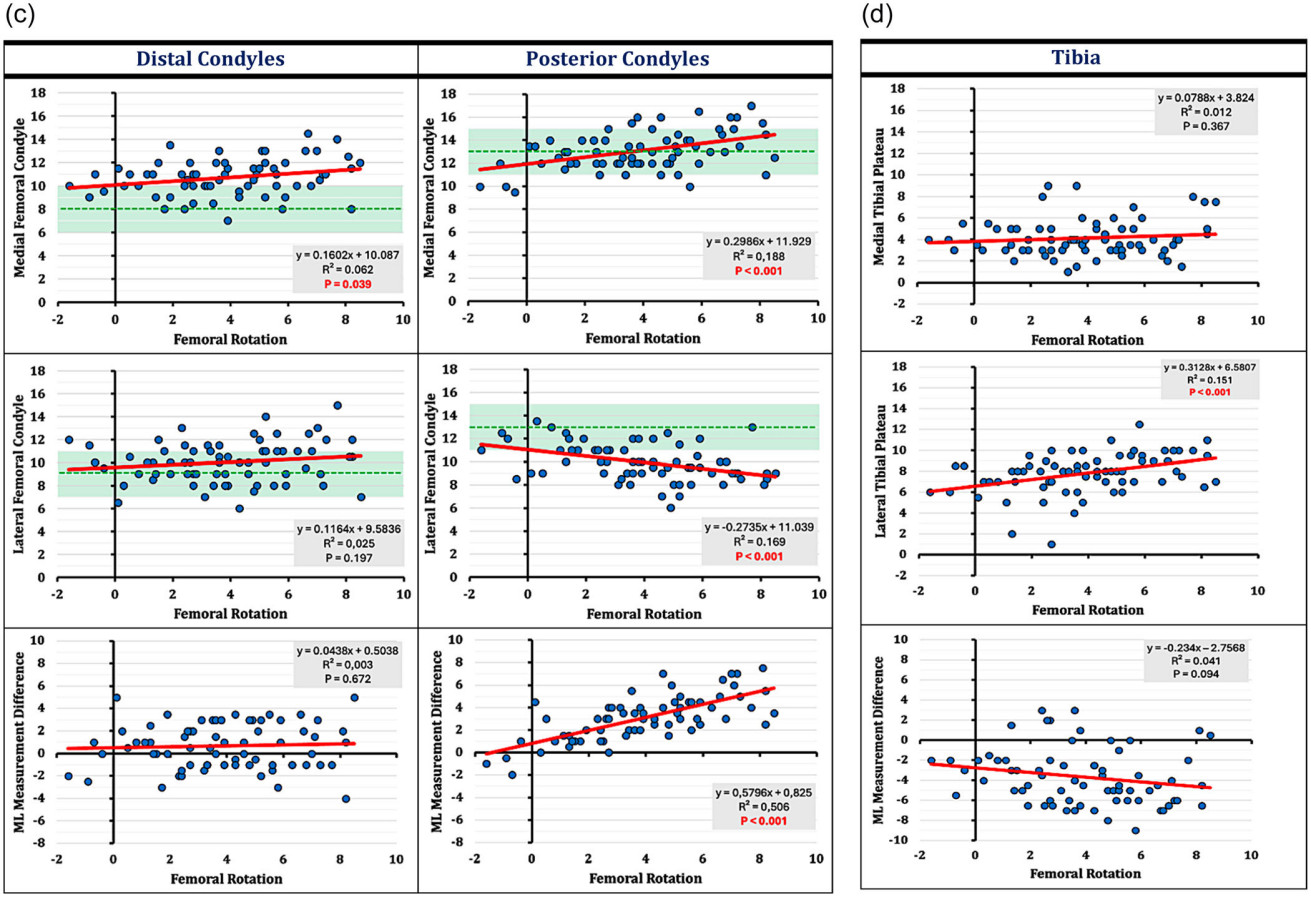

There were different modification‐patterns observed between the different measurement areas of the anterior trochlea. The oblique trochlea cuts were less affected by the FR. The trochlea groove (Figure 4b) showed non‐significant changes with a positive relation to external FR. The distal femur showed the same modification pattern on both condyles with a positive relation to external FR. The moderate ML differences of the distal femur throughout the range of rotation reflects the (tibia‐based FA) surgical technique used, where the distal femoral cut is defined mainly by the tibia irrespective of FR (Figure 4c,d). Finally, while the medial tibia cut was less affected by the FR (less than 1 mm changes), the lateral tibia showed increasing resection thicknesses approximately 3 mm with a positive weak correlation (*r* = 0.39, *p* < 0.001) to external FR (Figure 4c).

The results of the stuffing analysis including 759 measurements for the femur and 207 for each side of the trochlea (medial margin, groove, lateral margin) are summarised on the hybrid Table 4a,d. The overall femoral and trochlear stuffing incidence >2 mm was less than 40% in all cases. Finally, the lowest stuffing incidence was detected in FR of 2°.

**Table 4:** (a–d) Stuffing analysis of (a) the femur (includes all resections) within the whole range of motion of the knee for different femoral rotation groups (ER < 1°, 1°, 2°, 3°, ≥4°). The stuffing analysis of (b) the medial margin of the trochlea, (c) the trochlear groove, and (d) the lateral margin of the trochlea includes the anterior, oblique and distal resection planes.

(a)							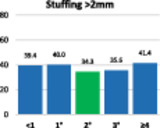	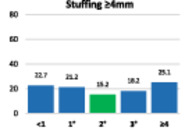
Femur	**ER**	**No**	**%**	M* ± SD	**>2 mm**	**≥4 mm**		
	<1°	69	9.1	2.3 ± 2.3	39.4	22.7		
	1°	88	11.6	2.1 ± 1.8	40.0	21.8		
	2°	103	13.6	1.8 ± 1.6	33.3	15.2		
	3°	135	17.8	1.9 ± 1.8	35.6	18.2		
	≥4°	364	47.9	2.3 ± 1.9	41.4	25.1		
	ALL	759	100	2.2 ± 1.9	39.5	22.2		
(b)							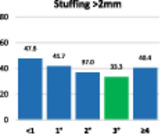	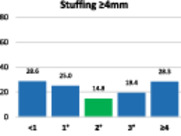
Trochlear medial margin	**ER**	**No**	**%**	M* ± SD	**>2 mm**	**≥4 mm**		
	<1°	21	10.1	3.1 ± 2.4	47.6	28.6		
	1°	24	11.6	2.1 ± 2.0	41.7	25.0		
	2°	27	13.0	1.8 ± 1.7	37.0	14.8		
	3°	36	17.4	1.8 ± 1.7	33.3	19.4		
	≥4°	99	47.8	2.4 ± 2.1	40.4	28.3		
	ALL	207	100	2.3 ± 2.0	39.6	24.6		
(c)							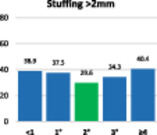	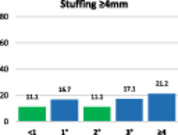
Trochlear groove	**ER**	**No**	**%**	M* ± SD	**>2 mm**	**≥4 mm**		
	<1°	21	10.1	1.6 ± 1.7	38.9	11.1		
	1°	24	11.6	1.8 ± 1.6	37.5	16.7		
	2°	27	13.0	1.7 ± 1.7	29.6	11.1		
	3°	36	17.4	2.0 ± 2.0	34.3	17.1		
	≥4°	99	47.8	1.9 ± 1.9	40.4	21.2		
	ALL	207	100	1.9 ± 1.8	37.7	18.4		
(d)							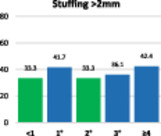	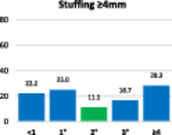
Trochlear lateral margin	**ER**	**No**	**%**	M* ± SD	**>2 mm**	**≥4 mm**		
	<1°	21	10.1	2.6 ± 2.0	33.3	22.2		
	1°	24	11.6	2.2 ± 1.7	41.7	25.0		
	2°	27	13.0	1.8 ± 1.5	33.3	11.1		
	3°	36	17.4	1.8 ± 1.6	36.1	16.7		
	≥4°	99	47.8	2.3 ± 1.9	42.4	28.3		
	ALL	207	100	2.2 ± 1.9	39.6	23.7		

*Note*: The green column on the bar charts indicates the lowest incidence (in some cases reached by two groups).

Abbreviations: ER, external rotation of the femur; SD, standard deviation.

*Mean ± standard deviation of absolute difference between implant thickness and resection thickness to assess stuffing regardless of direction (over vs. under).

## DISCUSSION

The most important outcome of this study was the fact that external FR of 2° using an off‐the‐shelf implant utilising a tibia‐based FA protocol was associated with the lowest incidence of stuffing >2 mm and ≥4 mm around the femur.

There was an overall moderate femoral anatomy modification with 61% below 2 mm and 78% below 4 mm changes. The most affected areas with the highest >2 mm stuffing incidence were the lateral trochlea facet (61%), the medial distal condyle (78%) and the lateral posterior condyle (64%). The overall stuffing incidence for >2 mm and >4 mm was higher in CAM than in TAM.

The main modification patterns observed were a TAM in extension with a mean understuffing of the lateral flange −3.2 mm ± 2.4 mm ([95% CI: −3.71 to −2.63], *p* < 0.001) and a mean elevation of the anterior groove +0.7 mm ± 3.1 mm ([95% CI: 0.22–1.32], *p* = 0.02), whereas in midflexion and deep flexion there was an understuffing of the groove −1.7 mm ± 2.4 mm ([95% CI: −1.85 to −0.93], *p* < 0.001) and −1.8 mm ± 2.5 mm ([95% CI: 1.25–2.44], *p* = *n.s*.) respectively. This indicated a slight shifting of the groove centre to a more anterior‐proximal position. CAM was associated with an understuffing of the medial distal condyle −3.6 mm ± 2.3 mm ([95% CI: −4.14 to −3.05], *p* < 0.001), reflecting a medial joint line elevation, and with an overstuffing of the lateral posterior condyle +3 mm ± 2.3 mm ([95% CI: 2.48–3.38], *p* > 0.001), corresponding to an increased lateral posterior femoral offset. The current CAM‐pattern shifted the cylindrical flexion‐extension axis of the knee into a more varus and external rotated orientation (towards mechanical alignment axes).

### Hypothesis no 1

The results of the current study, with an overall incidence of 39.6% and 37.7% induced stuffing > 2 mm around the femur and the full arc of the trochlea, could reject the 1^st^ hypothesis. This outcome is consistent with the literature [62]. Shatrov et al. used a single‐radius implant design employing the FA technique and found 44.3% incidence of stuffing > 2 mm on the trochlear groove in midflexion and deep flexion. Their TAM pattern varied with over‐ and understuffing found in 2.5% and 32% in midflexion, and 20.5% and 24.6% in deep flexion respectively. The current study, combining a multi‐radius implant design with FA found stuffing > 2 mm in 37% on the trochlear groove in midflexion and deep flexion. However, a different TAM pattern was observed with mainly mild groove understuffing in both, midflexion (−1.7 ± 2.4 mm) and deep flexion (−1.8 ± 2.5mm) (Figure 4b). Lyon's team reported a balanced anterior groove below 2 mm in all cases (100%). While both studies had equal mean values on the anterior groove (0.73 mm vs. 0.70 mm), the current cohort showed a much higher variance of distribution with a range of 10.5 mm (−5.5 to 5 mm) versus 4 mm (−2 to 2 mm) and a 60.1% incidence of balanced anterior groove. This can be explained by the differences in implant design and the wide inter‐rater variability of implant positioning within same knee phenotypes that occur under the umbrella of functional alignment.

### Hypotheses no 2 and 3

The current study could demonstrate the dynamic changes of the femoral anatomy in relation to progressive external rotation of the femoral component. Although the results are specific for the surgical technique and implant used it became transparent that femoral rotation affected mainly the posterior (*r* = 0.71, *p* < 0.001) and anterior (*r* = −0.40, *p* < 0.001) aspects of the joint and less the oblique and distal portion of the trochlea and the trochlear groove to its full range of flexion (Figure 4a–d). Thus, the second hypothesis could be confirmed. However, the posterior femoral condyles revealed a stronger correlation with the external FR than the anterior trochlea did. This reflects the very variable anatomy of the patellofemoral joint which is associated with anterior knee pain that is frequently seen in both, native and replaced knee joints [42]. Trochlea is much more difficult to reconstruct with a one‐size‐fits‐all implant than the condyles [25, 51, 62, 63]. Historically, from the “*Vertebrates*”, before approximately 400 million years, to the development of *human bipedalism* before 4 million years, and the modern form of “*Homo sapiens*”, femoral condyles have remained relatively unchanged in shape throughout human evolution, whereas the patellofemoral joint was established only 1.3 million years ago [9]. This delayed developmental background of the PFJ could explain why the trochlea is much more variable and does not relate to the rest tibiofemoral joint [4], while the condyles have a standard shape with an almost spherical form [42, 45].

New alignment philosophies aiming to resurface the knee [22] and therefore reduce the stuffing have failed to restore the trochlea anatomy accurately using MA‐designed implants [51]. Modern implants with trochlea friendly designs designated for KA‐use [24, 57], and novel new TKA prosthesis with individualised modular trochlea components have been introduced [27] to address this challenge.

Recent evidence suggests that personalised robotic‐assisted TKA utilising FA, through fine‐tuning of component position, can restore the trochlea groove more closely to the native knee when compared to other alignment philosophies [46, 63].

The current study using an off‐the‐shelf implant with FA technique could demonstrate that femoral rotation of 2° had the lowest stuffing rates around the whole femur and the trochlea through the full range of flexion (Table 4a,b). Thus, the third hypothesis could be rejected. This finding aligns with recent literature, where balanced trochlea's (<2 mm changes) were linked to a greater femoral external rotation (2.4°) than the unbalanced (1.2°) [62].

Rotation of the femur affects ligament function. Biomechanical studies have shown that external FR lengthens the medial patellofemoral ligament MPFL from 90° to 0° extension whereas non‐significant changes occur on the transverse fibres of the lateral retinaculum (LR) due to its attachments on the mobile ITB, who translates anteriorly in extension [14].

FR 0° restores accurately the posterior condylar anatomy in terms of offset and orientation but does not restore the trochlear anatomy when combined with the current available implants leading frequently to maltracking of the PFJ [70]. However, it seems that the greater patella tilt observed on static images in extension after KA has less clinical relevance [33]. A lateral patella shift, on the other hand, caused by a medialized implant trochlea groove in relation to the quadriceps vector, is associated with worst functional outcomes [24]. Furthermore, biomechanical data have shown that KA could reduce the quadriceps forces in flexion when compared with MA [64], which indicates the importance of restoring the distal joint line and posterior femoral offset to achieve improved PFJ function and pain. Thus, as PFJ forces increase with progressive flexion, reaching peak values in deep flexion [1, 8], it becomes more clear that optimising PFJ function after TKA is multifactorial beyond femoral rotation, and still far from being satisfactory [34].

### Patellofemoral overstuffing

Anterior overstuffing > 2 mm reduces the passive knee flexion to approximately 1.2° [5] to 3° [2] pro 2 mm increment and increases the PFJ articular forces in flexion up to 20% [16, 41, 68]. In more severe overstuffing +4 mm it stretches both retinacula (MPFL and LR) [13] and tensions the extensor mechanism 1.7 times more [60]. It is frequently considered cause for anterior knee pain [16]. Furthermore, PFJ overstuffing +2 mm may cause lateral patella tilt [40, 74], and lateral shift in early flexion which can trigger anterior knee pain [66].

﻿In a clinical study, overstuffed PFJ > 1 mm were associated with a longer recovery time (*p* < 0.05), and a lower flexion activity at 2 years follow‐up (*p* < 0.001). No significant differences were found in VAS at 2 years (*p* > 0.05) [71].

Although there is multiple biomechanical evidence supporting the negative effect of PFJ overstuffing, there is a lack of consensus across cohort studies on its clinical relevance and a universal definition for stuffing with clear boundaries is still missing [16].

### Patellofemoral understuffing

Maintenance of the physiological native anterior offset without overstuffing is important to keep extensor mechanism stretched and functionally competent [1].

PFJ understuffing reduces the moment arm of the extensor mechanism leading to overload of the quadriceps. 4 mm of understuffing causes quadriceps stressing 2.1 times more performing the same functions, leading to fatigue and weakness [8, 65]. A reduced moment arm of the extensor mechanism can have trochlear (reduced anterior offset), condylar (reduced posterior offset) or implant‐related causes (J‐curved femoral profile) [13, 41].

Understuffing of the lateral flange is a result of both implant alignment and implant design. The missing support of the otherwise elevated native lateral trochlea flange may pose a further risk for patellofemoral instability.

### Condylar stuffing

In the current study, the medial distal condyle was understuffed (−3.6 ± 2.3mm). Understuffing the medial distal condyle > 2 mm has several potential risks: (1) It can change the isometry of the MCL with less tension in extension than in flexion which may cause midflexion instability, pain and stiffness. (2) The medial retinaculum slackens in flexion boosting a more lateralized patella tracking. Clinical evidence supports that raising the medial joint line has a negative effect on patient reported outcomes [43, 49].

Overstuffing of the lateral distal condyle (also seen when varising joint lines) is associated with increased risk for stretching the ITB close to extension [47] and the lateral retinaculum in flexion [52, 67] triggering anterolateral knee pain, patella maltracking and stiffness of the extensor mechanism.

On the posterior aspect, a well restored medial condyle (−0.2 ± 3.2 mm) and an overstuffed lateral condyle (3.0 ± 2.3 mm) were observed in the current study. Overstuffing of the lateral posterior condyle >2 mm is associated with poorer functional outcomes: [39, 50, 72] (1) It stretches the posterior lateral structures affecting the pivoting of the femur and the extension of the knee, (2) It increases the level arm of the extensor mechanism and stretches the quadriceps affecting the flexion of the knee, (3) an LCL imbalance (overstretching in flexion, normal in extension) can compromise ligament function and cause midflexion instability. (4) A possible stretching of the popliteal tendon can also lead to posterolateral and rotational pain with functional restrictions.

This study used a posterior reference system. Compared with it, anterior reference guides are associated with reduced anterior and posterior offsets [44], which increases the flexion gap requiring ligament balancing or recuts, and stresses more the extensor mechanism.

Height (mm) and orientation (°) are two different dimensions. Groove height can be restored even in different trochlear orientations. Peripheral bony structures are more sensitive to orientational changes in both, the coronal and axial planes (Figure 4a–d). Modification patterns with crossing implant thickness line and turn from overstuffing to understuffing or vice versa remain usually within the balanced zone of ±2 mm (e.g., the medial trochlear margin in extension and midflexion, and medial posterior condyle in flexion). Modification patterns with stuffing > 2 mm have a unidirectional character (e.g., lateral trochlear facet in extension, medial distal condyle in extension, lateral posterior condyle in flexion).

As at the current state of knowledge and implant availability, restoration of the anterior space is unpredictable and clinically relevant pathologies are more prone in flexion, where the distal joint line plays a major role, it makes sense to prioritise the condylar anatomy and the gap balance of the knee. However, whether prioritising condylar reconstruction ignoring the trochlea or balancing implant position between trochlea and condylar articular surfaces to reduce overall stuffing on both sides is superior over the other, there is no clear consensus in the literature and therefore still potential limitations and drawbacks for both techniques [4]. Thus, aiming to restore the articular surfaces (TFJ and PFJ) within 2 mm of height changes and 2° of orientational changes in all resection planes seems to be a reasonable target for improved functional outcomes.

### Strengths and limitations

This is the first study using real‐time caliper measurements to assess trochlea and condylar anatomy modification patterns and stuffing incidences around the femur after Ra TKA using a tibia‐based FA protocol. Furthermore, as personalised TKA should not be just on coronal alignment but also on other planes [55], this study demonstrates the relation between FR and femoral stuffing on 11 different areas around the knee.

An imageless robotic system was used in the current study. Although differences between imageless versus image‐based RaTKA have been described [18], it's high accuracy in terms of degree difference between planed and measured resection angles is documented in multiple publications [3, 58, 59, 69, 73]. Using the same robotic system Seidenstein et al. found an accuracy in resection angles 0.6° ± 0.4° [59]. Finally, caliper resolution is 0.5 mm [23].

Limitations of the study is a possible type‐II‐error due to the increased variability of the trochlea and the reduced sample sizes of the subgroups that might affect the power of the study with pseudo‐negative values. However, power‐analyses for each hypotheses tested suggested sufficient sizes. The use of a PS design led to difficulties in the measurement of the central oblique trochlea via caliper because of the interfering PS box, and the values were given by the manufacturer. Furthermore, the outcomes of the current study are representative only of the tested arthroplasty components and not for other implants with different design properties and therefore cannot be generalised. However, the lower stuffing rates after 2° external FR found in the current study, could be also demonstrated with different implant designs using same technique [62]. Finally, no data regarding the incidence and extent of ligament releases were documented in the study. This might have influenced the bony resections. The clinical relevance of these findings especially in relation with modern alignment techniques is unknown and therefore a subject for further clinical studies.

## CONCLUSION

While FA with off‐the‐shelf implants leads to typical TAM and CAM patterns, a femoral rotation of 2° may induce the lowest rates of stuffing around the femur without influencing the trochlear groove to its full range of flexion. However, the highly variable PFJ makes restoration of the anterior knee space with current implants unpredictable and is related with condylar anatomy compromises. Hence, further studies are required to assess the clinical significance of these findings and to define the clinically relevant boundaries for stuffing around the knee. As functionally aligned implant positions vary considerably, stuffing analyses on pure MA and KA techniques with defined rotational profiles of the components in relation to the native condylar anatomy might provide further clarity to this topic. New implant designs adaptable to personalised TKA are needed.

## AUTHOR CONTRIBUTIONS

**Alexander Maslaris**: Study design; data analysis; literature review; manuscript writing; figures and tables; manuscript revision. **Eustathios Kenanidis**: Surgeries; data analysis; statistics and supervision of data collection. **Nikolaos Mylonakis** and **Zakareya Gamie**: Data collection; data analysis. **Abtin Alvand**: Proof reading. **William Jackson**: Literature review; review manuscript. **Andrew J. Price**: Review manuscript. **Eleftherios Tsiridis**: Surgeries, PI.

## CONFLICT OF INTEREST STATEMENT

The authors declare no conflicts of interest.

## ETHICS STATEMENT

Obtained prior to study begin ﻿(IRB No. 4/13.02.2024).

## Data Availability

The data that support the findings of this study are available from the corresponding author upon reasonable request.
